# Supplementary material for: Nitrogen Source Preference and Growth Carbon Costs of Leucaena leucocephala (Lam.) de Wit Saplings in South African Grassland Soils
Source: Plants (Basel). 2021 Oct 21;10(11):2242. doi: 10.3390/plants10112242 (PMC8621804; doi:10.3390/plants10112242)
Supplement: Supplementary file 1 [file plants-10-02242-s001.zip › plants-1372784-supplementary.pdf]

Supplementary table 1. Soil characteristics determined from the Veld Fertilizer Trial at Ukulinga Experimental Farm, South Africa. Values represent the means ± SE, based on n = 4. Significant differences among treatments ($p < 0.05$) are denoted by different superscript letters.

	Treatment trials					
**Parameter**	**N1**	**N2**	**N3**	**N1+P**	**N2+P**	**N3+P**
**Soil characteristics**						
Leco N concentration (μmol N g^{-1})	$0.27±0.01^{a}$	$0.32±0.01^{c}$	$0.31±0.00^{bc}$	$0.31±0.01^{bc}$	$0.28±0.01^{ab}$	$0.29±0.01^{ac}$
P concentration (μmol P g^{-1})	$0.07±0.013^{a}$	$0.16±0.05^{bc}$	$0.10±0.01^{ab}$	$0.21±0.01^{c}$	$0.20±0.04^{c}$	$0.22±0.04^{c}$
K concentration (μmol K g^{-1})	$3.81±0.58^{b}$	$3.07±0.58^{ab}$	$4.29±0.81^{b}$	$3.14±0.27^{ab}$	$1.74±1.45^{a}$	$1.45±0.14^{a}$
Exchange acidity	$0.22±0.06^{a}$	$0.75±0.30^{b}$	$1.57±0.23^{c}$	$0.13±0.01^{a}$	$0.23±0.05^{a}$	$1.02±0.23^{bc}$
Moisture factor	$1.07±0.03^{a}$	$1.08±0.00^{b}$	$1.08±0.00^{b}$	$1.08±0.00^{b}$	$1.08±0.00^{ab}$	$1.08±0.00^{ab}$
pH (KCl)	$4.67±0.07^{bc}$	$4.46±0.09^{ab}$	$4.12±0.04^{a}$	$4.77±0.04^{bc}$	$5.01±0.37^{c}$	$4.18±0.04^{ab}$
pH (H_20)	$5.63±0.07^{c}$	$5.45±0.10^{bc}$	$4.61±0.14^{a}$	$5.72±0.06^{c}$	$5.86±0.27^{c}$	$4.91±0.12^{ab}$

Supplementary table 2. The molecular identification of soil nitrogen fixing bacteria and plant root nodule isolated nitrogen fixing bacteria in *L. leucocephala* saplings grown in Veld Fertilizer Trial soils obtained from Ukulinga Experimental Farm, South Africa.

Nitrogen fixing bacteria			Treatment (s)
Strains	Accession No.	Similarity (%)	
Veld Fertilizer Trial soil isolated N-fixing bacteria			

Caulobacter rhizosphaerae strain IMCC34905	MK 138628	97.38	N1, N2, N3, N1 + P, N2 + P and N3 + P
Sphingomonas sp. N-9	LC 101917	97.82	N1, N2, N3
Burkholderia contaminans strain J8A6SARS	MT 409575	99.01	N1 + P, N2 +P and N3 + P
L. leucocephala saplings root nodule isolated N-fixing bacteria			
Rhizobium sp.	KC355318.1	98.01	N2 + P
Mesorhizobium sp. strain BwlS3- 3	KX 959573	98.00	N2 + P
